# Supplementary material for: Optimization and Standardization of Human Saliva Collection for MALDI-TOF MS
Source: Diagnostics (Basel). 2021 Jul 21;11(8):1304. doi: 10.3390/diagnostics11081304 (PMC8392517; doi:10.3390/diagnostics11081304)
Supplement: Supplementary file 1 [file diagnostics-11-01304-s001.zip › diagnostics-1255213-supplementary.pdf]

diagnostics

MDPI

Supplementary Material

Optimization and Standardization of Human Saliva Collection for MALDI-TOF MS

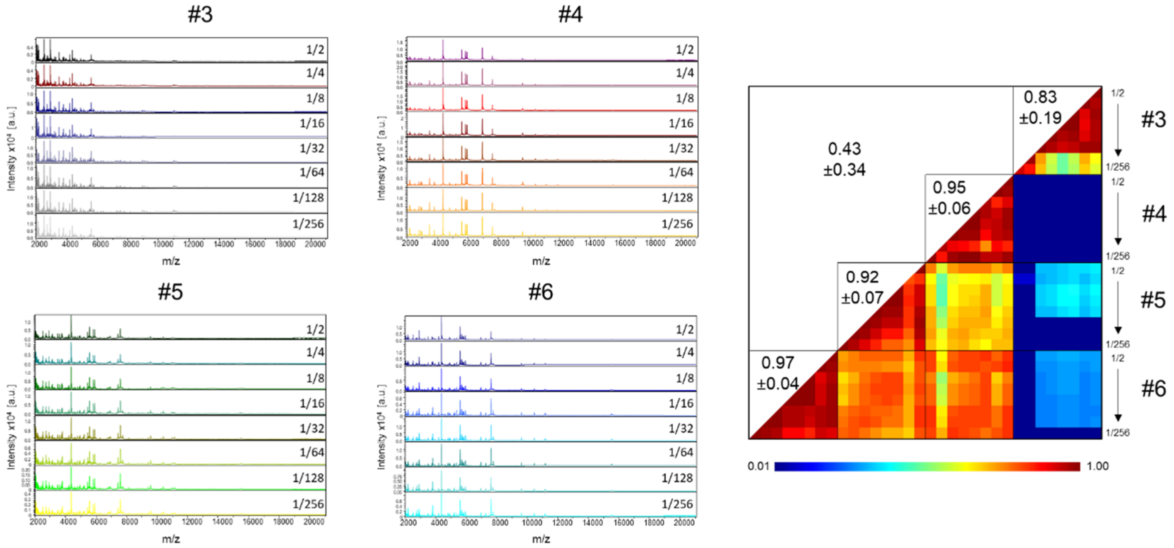

Figure S1. MS profiles of serial dilution of saliva from healthcare workers. (**a**) Representative MS spectra of serial dilution of saliva, collected in plastic tube, prepared according to protocol A from four individuals. The dilution rate is indicated at the right part of each profile. (**b**) Composite correlation index (CCI) matrix representing the levels of MS spectra reproducibility between saliva samples. CCI are expressed as the mean ± standard deviation. #3 to #6: healthcare workers.

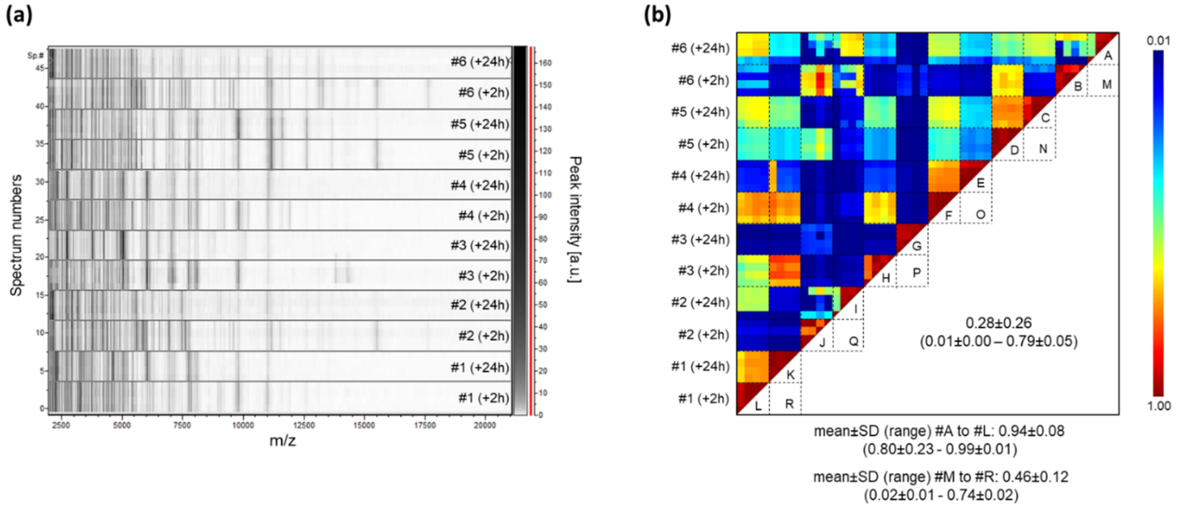

Figure S2. Effect of daily storing on stability of salivary MS profiles. (**a**) Gel view of saliva MS spectra collected with Salivette and stored at 4 °C during 2 and 24 h. The four replicates loaded on the MS plate for each individual per condition are presented. #1 to #6: healthcare workers. (**b**) Composite correlation index (CCI) matrix representing the levels of MS spectra reproducibility between saliva samples per individual according to storing duration. The levels of MS spectra reproducibility are indicated in red and blue revealing relatedness and incongruence between spectra, respectively. CCI are expressed as the mean ± standard deviation. #1 to #12: healthcare workers. #A to #L: assessment of individual MS spectra reproducibility. #M to #R: assessment of MS spectra reproducibility at one day interval from paired samples per individual.

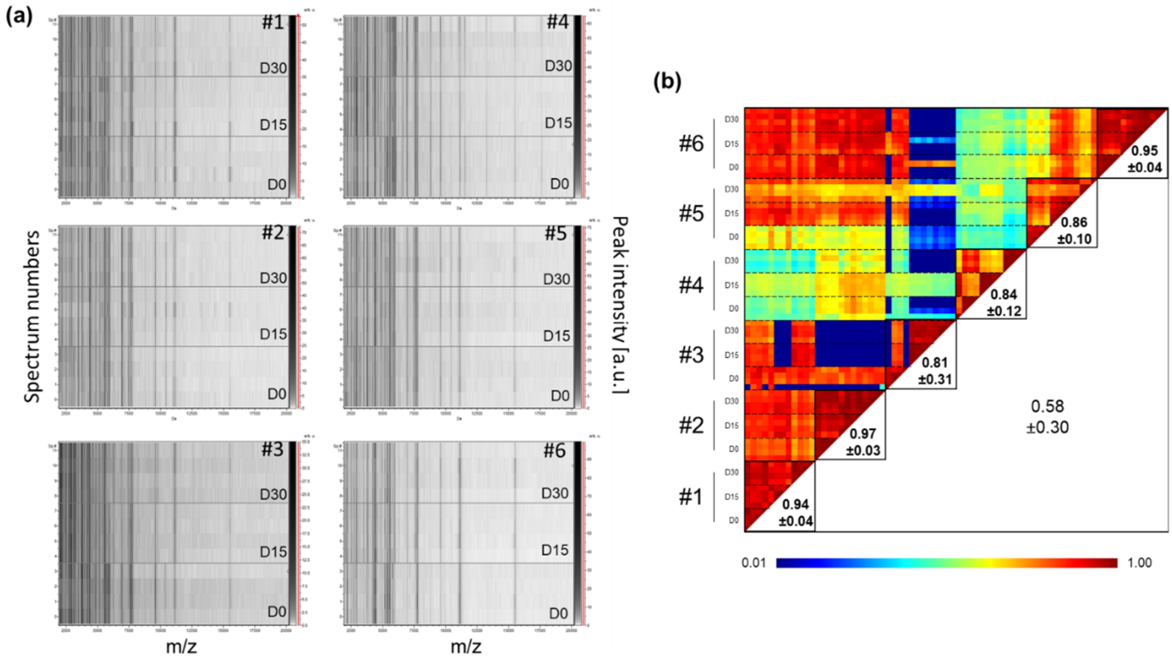

Figure S3. Stability of saliva MS profiles from same individuals along one month. (**a**) Gel view of saliva MS spectra collected with Salivette from 4 individuals at 3 time points with two weeks of interval (Day, D0, D15 and D30). The four replicates loaded on the MS plate for each individual per collection time point are presented. #1 to #4: healthcare workers. (**b**) Composite correlation index (CCI) matrix representing the levels of MS spectra reproducibility between saliva samples from the same individual collected at different time points. The levels of MS spectra reproducibility are indicated in red and blue revealing relatedness and incongruence between spectra, respectively. The values correspond to the mean coefficient correlation and respective standard deviations obtained.
